# Genome-wide mRNA expression analysis of peripheral blood from patients with obsessive-compulsive disorder

**DOI:** 10.1038/s41598-018-30624-1

**Published:** 2018-08-22

**Authors:** Yuqing Song, Yansong Liu, Panpan Wu, Fuquan Zhang, Guoqiang Wang

**Affiliations:** 1 Peking University Sixth Hospital (Institute of Mental Health), Key Laboratory of Mental Health, Ministry of Health (Peking University), National Clinical Research Centre for Mental Disorders (Peking University Sixth Hospital), Beijing, 100191 China; 2 Department of Clinical Psychology, Suzhou Psychiatric Hospital, The Affiliated Guangji Hospital of Soochow University, Suzhou, 215137 Jiangsu China; 3 Wuxi Mental Health Centre, Nanjing Medical University, Wuxi, 214151 Jiangsu China

## Abstract

The onset of obsessive-compulsive disorder (OCD) involves the interaction of heritability and environment. The aim of this study is to identify the global messenger RNA (mRNA) expressed in peripheral blood from 30 patients with OCD and 30 paired healthy controls. We generated whole-genome gene expression profiles of peripheral blood mononuclear cells (PBMCs) from all the subjects using microarrays. The expression of the top 10 mRNAs was verified by real-time quantitative PCR (qRT-PCR) analysis. We also performed an enrichment analysis of the gene ontology (GO) and Kyoto Encyclopaedia of Genes and Genomes (KEGG) annotations of the differentially expressed mRNAs. We identified 51 mRNAs that were significantly differentially expressed between the subjects with OCD and the controls (fold change ≥1.5; false discovery rate <0.05); 45 mRNAs were down-regulated and 6 mRNAs were up-regulated. The qRT-PCR analysis of 10 selected genes showed that they were all up-regulated, which was opposite to the results obtained from the microarrays. The GO and KEGG enrichment analysis showed that ribosomal pathway was the most enriched pathway among the differentially expressed mRNAs. Our findings support the idea that altered genome expression profiles may underlie the development of OCD.

## Introduction

Obsessive-compulsive disorder (OCD) is characterized by recurrent intrusive thoughts or images (obsessions) and/or ritualized behaviours (compulsions) that cause marked distress and impairment to a person^1^. About 1–3% of the general population suffers from OCD^2–5^ and the symptoms appear before age 25 years in about two-thirds of affected persons, with the mean age of onset about 20 years^2,4^. Both environmental and genetic factors are deemed to play important roles in the aetiology of OCD and genetic factors account for about 45–65% of variance in OCD if the disorder occurs in childhood^6^. Despite the great progress made in understanding the pathogenesis of the disorder, the genetic causes of OCD remain elusive. This may be because the aetiology of OCD is complex and probably related with multiple independent and interacting genetic factors.

The completion of the Human Genome Project and the development of genome-wide screening have made microarray technology an important tool to study genetic effects on the aetiology of psychiatric disorders^7^. Previous studies have found genes in blood and brain tissues share similar expression patterns^8,9^, and it is easier to measure gene expression in blood because it can be obtained with minimal invasiveness. Microarray approaches have been widely used to investigate neuropsychiatric disorders such as schizophrenia^10^, bipolar disorder^11^, and major depressive disorder (MDD)^12^. In addition, microarray data have been used to find novel genes and pathways that may be related with the aetiology of psychiatric disorders^7^. However, to our knowledge, microarrays have not been applied to investigate gene expression in the peripheral blood of OCD patients. In this study, we aimed to detect genes (mRNAs) that were differentially expressed between patients with OCD and healthy controls using microarray technology. We also performed an enrichment analysis of the gene ontology (GO) terms and Kyoto Encyclopaedia of Genes and Genomes (KEGG) pathways assigned to the genes to investigate the functions of the differentially expressed mRNAs.

## Results

### Differentially expressed mRNAs

We used microarrays in a genome-wide scan of mRNAs from peripheral blood mononuclear cells (PBMCs) of 30 patients with OCD and 30 paired healthy controls.

**Figure 1:**
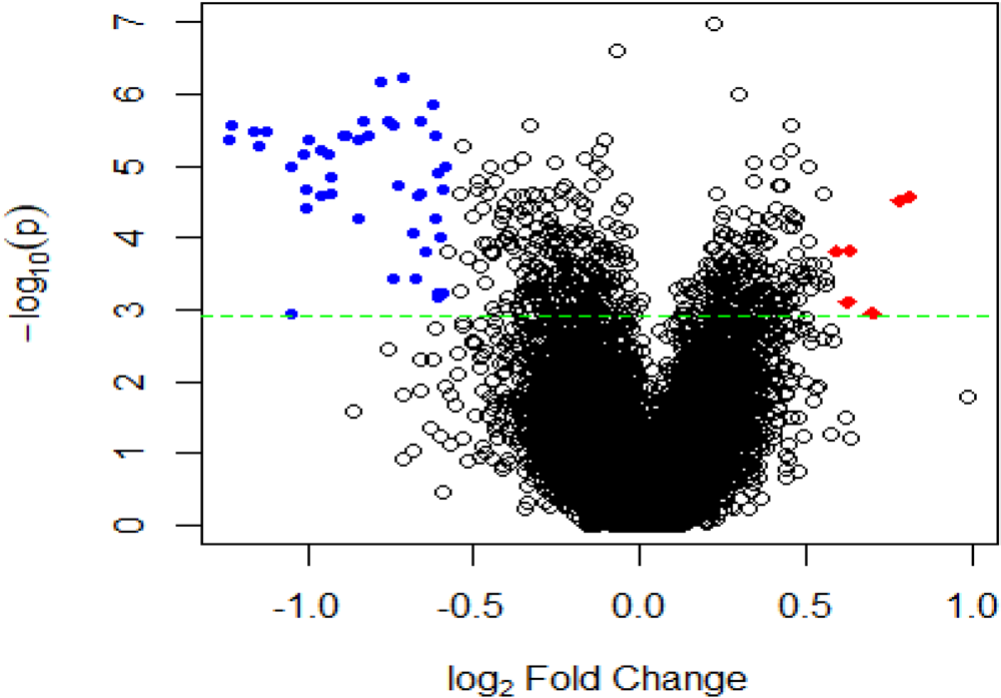
Volcano plot of changes in the whole-genome gene expression profiles of peripheral blood mononuclear cells between OCD patients and healthy controls. A total of 51 significantly differentially expressed mRNAs with fold change ≥1.5 and Bonferroni-adjusted *p*-value < 0.05 were detected. Blue dots indicate the 45 down-regulated genes, red dots indicate the 6 up-regulated genes. The horizontal green line is the negative logarithm of the Bonferroni-adjusted *p*-value threshold.

**Figure 2:**
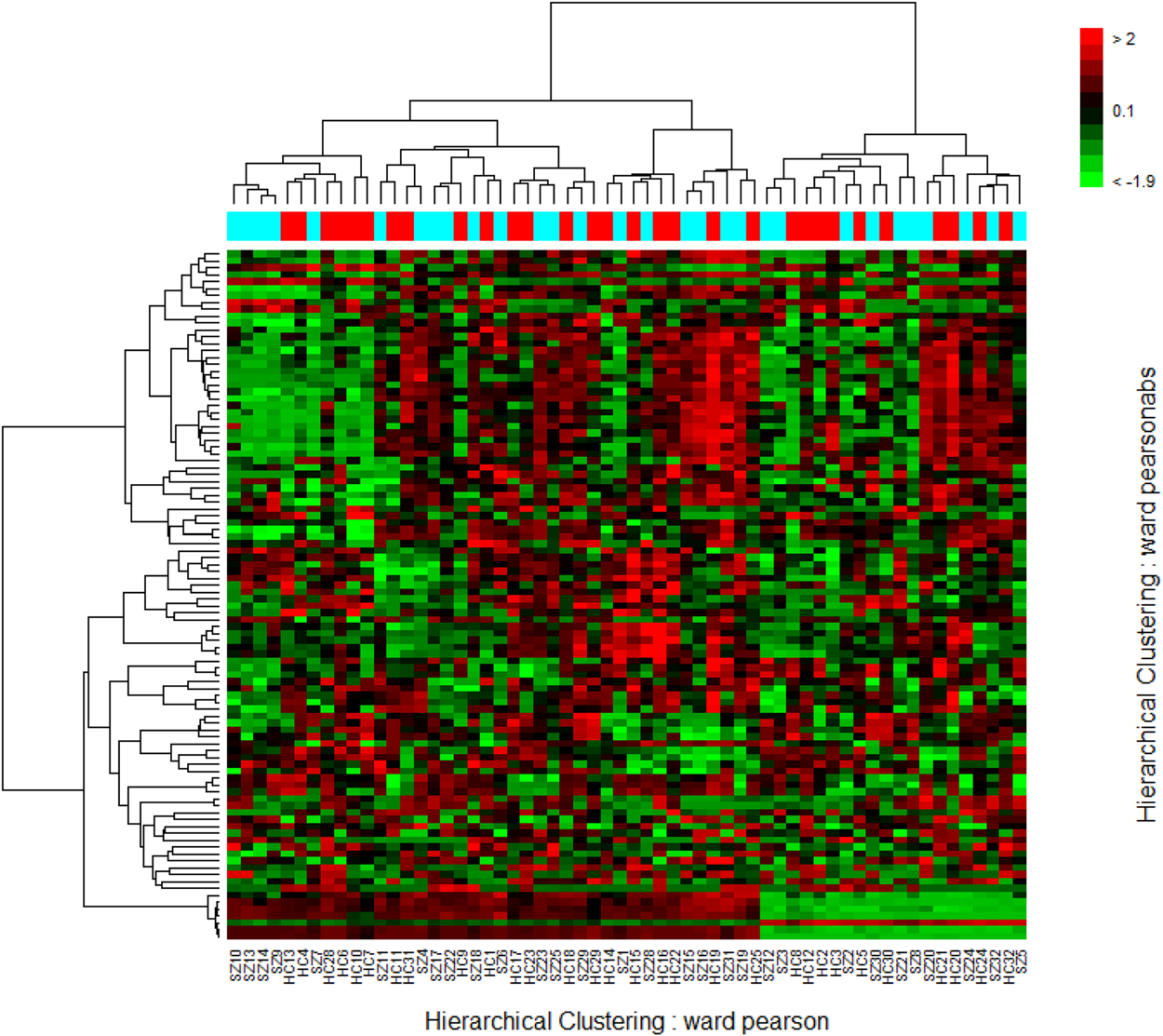
Heatmap of the top 100 differentially expressed genes that can distinguish OCD patients and healthy controls obtained by hierarchical cluster analysis.

We detected a total of 51 differentially expressed mRNAs with fold change ≥1.5 and false discovery rate <0.05; 45 were down-regulated and 6 were up-regulated (Table 1, Fig. 1). The hierarchical cluster analysis showed that the samples separated into distinct patient and control groups (Fig. 2).

**Table 1:** All the differentially expressed genes (Benjamini-Hochberg adjusted *p*-value < 0.05) detected between the OCD patients and healthy controls with current NCBI Entrez gene records.

	Gene Symbol	Gene Name	Chromosome	regulation	p Value	Fold change	FDR
1	RPL27	ribosomal protein L27	chr17	down	6.91E-07	0.582762948	0.003187
2	RPS18	ribosomal protein S18	chr6	down	5.72E-07	0.610788004	0.003187
3	RPL26	ribosomal protein L26	chr17	down	4.42E-06	0.42481714	0.003546
4	COMMD6	COMM domain containing 6	chr13	down	2.76E-06	0.427294299	0.003546
5	RPL34	ribosomal protein L34	chr4	down	3.24E-06	0.445884809	0.003546
6	RPS7	ribosomal protein S7	chr2	down	3.24E-06	0.458631749	0.003546
7	RPL31	ribosomal protein L31	chr2	down	4.42E-06	0.501410942	0.003546
8	RPL39	ribosomal protein L39	chrX	down	3.79E-06	0.539614189	0.003546
9	TOMM7	translocase of outer mitochondrial membrane 7 homolog (yeast)	chr7	down	3.79E-06	0.542138227	0.003546
10	EEF1B2	eukaryotic translation elongation factor 1 beta 2	chr2	down	4.42E-06	0.556688907	0.003546
11	RPS15A	ribosomal protein S15a	chr16	down	2.35E-06	0.562020756	0.003546
12	RGS4	regulator of G-protein signaling 4	chr1	down	3.79E-06	0.566413939	0.003546
13	RPL35	ribosomal protein L35	chr9	down	2.35E-06	0.59062958	0.003546
14	RPS29	ribosomal protein S29	chr14	down	2.76E-06	0.596378692	0.003546
15	SNRPD2	small nuclear ribonucleoprotein D2 polypeptide 16.5 kDa	chr19	down	2.35E-06	0.631475109	0.003546
16	RPL35A	ribosomal protein L35a	chr3	down	1.42E-06	0.648175622	0.003546
17	RPL6	ribosomal protein L6	chr12	down	3.79E-06	0.652710016	0.003546
18	RPS24	ribosomal protein S24	chr10	down	5.14E-06	0.450604138	0.003796
19	RPL17	ribosomal protein L17	chr18	down	5.97E-06	0.513045476	0.003935
20	RPL23	ribosomal protein L23	chr17	down	6.92E-06	0.495724775	0.004254
21	RPS17	ribosomal protein S17	chr15	down	6.92E-06	0.522941699	0.004254
22	RPL41	ribosomal protein L41	chr12	down	1.06E-05	0.483001202	0.004773
23	RPL11	ribosomal protein L11	chr1	down	1.06E-05	0.664963757	0.004773
24	RPS21	ribosomal protein S21	chr20	down	1.22E-05	0.656973082	0.005226
25	PFDN5	prefoldin subunit 5	chr12	down	1.40E-05	0.523857014	0.005855
26	RPL21	ribosomal protein L21	chr13	down	1.82E-05	0.603359786	0.006472
27	RPL7	ribosomal protein L7	chr8	down	2.08E-05	0.496959421	0.006716
28	RPS27	ribosomal protein S27	chr1	down	2.37E-05	0.525259984	0.006716
29	COX7C	cytochrome c oxidase subunit VIIc	chr5	down	2.37E-05	0.631620933	0.006716
30	NDUFA4	NADH dehydrogenase (ubiquinone) 1 alpha subcomplex, 4, 9 kDa	chr7	down	2.08E-05	0.661901404	0.006716
31	UQCRB	ubiquinol-cytochrome c reductase binding protein	chr8	down	2.69E-05	0.51308111	0.006985
32	TAS2R46	taste receptor, type 2, member 46	chr12	up	2.69E-05	1.755854399	0.006985
33	CD52	CD52 molecule	chr1	down	2.69E-05	0.630299847	0.006985
34	OCR1	ovarian cancer-related protein 1	chr1	up	3.05E-05	1.721799154	0.007602
35	RPS3A	ribosomal protein S3A	chr4	down	3.90E-05	0.496805364	0.008573
36	RPL9	ribosomal protein L9	chr4	down	5.59E-05	0.554658337	0.010214
37	NDUFA1	NADH dehydrogenase (ubiquinone) 1 alpha subcomplex, 1, 7.5 kDa	chrX	down	5.59E-05	0.651853357	0.010214
38	XRCC6BP1	XRCC6 binding protein 1	chr12	down	8.86E-05	0.621641741	0.012966
39	HINT1	histidine triad nucleotide binding protein 1	chr5	down	9.90E-05	0.659365125	0.013141
40	KLRB1	killer cell lectin-like receptor subfamily B, member 1	chr12	down	0.000153	0.640029477	0.015753
41	MANSC1	MANSC domain containing 1	chr12	up	0.000153	1.552073368	0.015753
42	TAS2R30	taste receptor, type 2, member 30	chr12	up	0.000153	1.507579076	0.015753
43	RPL22L1	ribosomal protein L22-like 1	chr3	down	0.00038	0.598540111	0.024601
44	COMMD8	COMM domain containing 8	chr4	down	0.00038	0.626133659	0.024601
45	NDUFA5	NADH dehydrogenase (ubiquinone) 1 alpha subcomplex, 5, 13 kDa	chr7	down	0.00038	0.627359758	0.024601
46	GZMA	granzyme A (granzyme 1, cytotoxic T-lymphocyte-associated serine esterase 3)	chr5	down	0.000608	0.656586312	0.029848
47	RPS27L	ribosomal protein S27-like	chr15	down	0.000608	0.662613945	0.029848
48	MRPS28	mitochondrial ribosomal protein S28	chr8	down	0.000667	0.656263551	0.030893
49	FBXL13	F-box and leucine-rich repeat protein 13	chr7	up	0.000798	1.545602201	0.033757
50	ZNF721	zinc finger protein 721	chr4	down	0.001131	0.483970074	0.040059
51	CACNB4	calcium channel, voltage-dependent, beta 4 subunit	chr2	up	0.001131	1.628645369	0.040059

### Functional annotation

The GO functional enrichment analysis revealed 23 of the 51 significantly differentially expressed mRNAs were enriched in ribosomal protein terms, including cellular protein metabolic process, endocrine pancreas development, viral transcription, viral infectious cycle, viral reproduction, gene expression, translation, and RNA binding. The genes encoding NDUFA4 (NADH dehydrogenase (ubiquinone) 1alpha subcomplex), NDUFA5, and NDUFA1 were significantly enriched in mitochondrial electron transport and NADH to ubiquinone, and the genes encoding NDUFA4, COX7C (cytochrome c oxidase subunit VIIc), and UQCRB (ubiquinol-cytochrome c reductase binding protein) were significantly enriched in hydrogen ion transmembrane transport.

The KEGG pathway analysis of the 51 significantly differentially expressed mRNAs also identified 23 mRNAs that were enriched in ribosome; *NDUFA4*, *NDUFA5*, *COX7C*, *NDUFA1*, and *UQCRB*, which were significantly enriched in Parkinson’s disease, Alzheimer’s disease, Huntington’s disease, and non-alcoholic fatty liver disease, and *COX7C*, *CACNB4* (calcium channel, voltage-dependent, beta 4 subunit), and *UQCRB*, which were significantly enriched in cardiac muscle contraction (Table 2).

**Table 2:** Functional categories and biological function annotations based on gene ontology (GO) terms and KEGG pathways.

Category	Functions Annotation	p-Value	Molecules
KEGG analysis	Ribosome	8.15E-31	RPL35A, RPL17, RPL35, RPS15A, RPS27L, RPL22L1, RPL39, RPS7, RPS27, RPS29, RPL41, RPL7, RPL23, RPS17, RPS3A, RPL31, RPL6, RPL21, RPL9, RPL34, RPL11, RPS21, RPS24
	Oxidative phosphorylation	0.003482	NDUFA4, NDUFA5, COX7C, NDUFA1, UQCRB
	Parkinson’s disease	0.004403	NDUFA4, NDUFA5, COX7C, NDUFA1, UQCRB
	Non-alcoholic fatty liver disease (NAFLD)	0.005477	NDUFA4, NDUFA5, COX7C, NDUFA1, UQCRB
	Alzheimer’s disease	0.00796	NDUFA4, NDUFA5, COX7C, NDUFA1, UQCRB
	Huntington’s disease	0.012589	NDUFA4, NDUFA5, COX7C, NDUFA1, UQCRB
	Cardiac muscle contraction	0.04943	COX7C, CACNB4, UQCRB
Go analysis	Ribosome	2.57E-29	RPL35A, RPL17, RPL35, RPS15A, RPS27L, RPL22L1, RPL39, RPS7, RPS27, RPS29, RPL41, RPL7, RPL23, RPS17, RPS3A, RPL31, RPL6, RPL21, RPL9, RPL34, RPL11, RPS21, RPS24
	mitochondrial electron transport, NADH to ubiquinone	0.006979	NDUFA4, NDUFA5, NDUFA1
	hydrogen ion transmembrane transport	0.010652	NDUFA4, COX7C, UQCRB
	mitochondrial electron transport, cytochrome c to oxygen	0.050016	NDUFA4, COX7C

Significant biological pathways with two or more differentially expressed mRNAs representing each function are shown.

### Real-time quantitative PCR (qRT-PCR) validation

To validate the results of the microarray analysis, we chose 10 differently expressed mRNA transcripts for validation by qRT-PCR, namely, *RPS3A*, *RPL34*, *RPS24*, *RPL23*, *RPS7*, *RPL41*, *RPL7*, *RPL26*, *ZNF721*, and *COMMD6*. The qRT-PCR results showed that *RPL34* was down-regulated consistent with the microarray analysis, whereas *RPS3A*, *RPS24*, *RPL23*, *RPS7*, *RPL41*, *RPL7*, and *RPL26* were up-regulated (Table 3). *ZNF721* and *COMMD6* were not found to be differently expressed between OCD and healthy controls by qRT-PCR (Table 3).

**Table 3:** The mRNA expression levels in OCD patients and healthy controls by qRT-PCR.

	Gene Symbol	OCD (n = 26)	Healthy controls (n = 26)	Regulation	P value	Fold change	FDR
1	RPS7	5.33 ± 3.36	1.20 ± 1.11	up	2.576E-07	4.457	2.58E-06
2	RPS3A	4.23 ± 3.98	0.84 ± 0.70	up	8.292E-05	5.066	0.0004
3	RPL34	0.14 ± 0.11	0.33 ± 0.26	down	0.001	0.422	0.002
4	RPS24	30.01 ± 26.28	9.42 ± 14.64	up	0.001	3.188	0.002
5	RPL23	13.64 ± 10.98	6.53 ± 9.00	up	0.014	2.09	0.018
6	RPL41	5.60 ± 5.33	1.92 ± 3.00	up	0.003	2.913	0.004
7	RPL7	186.69 ± 251.24	23.61 ± 26.98	up	0.002	7.909	0.003
8	RPL26	7.22 ± 7.88	1.45 ± 2.05	up	0.001	4.971	0.002
9	ZNF721	0.73 ± 0.71	0.77 ± 0.81	—	0.858	0.951	0.858
10	COMMD6	0.45 ± 0.48	0.61 ± 0.58	—	0.299	0.746	0.332

## Discussion

To the best of our knowledge, this is the first study to reveal differentially expressed genes between patients with OCD and healthy controls using mRNA microarray technology. We found 45 mRNAs that were down-regulated and 6 mRNAs that were up-regulated in patients with OCD.

Previous studies have indicated that important genes involved in the pathophysiology of OCD were related to serotonin, dopamine, and glutamate systems^13–15^; however, we did not detect these genes in the present study. These discrepancies may be explained by the different research methods that were used. In previous studies, candidate gene approaches were used to explore OCD-related genes^13–15^, whereas we used microarrays to detect genes related to OCD^16^. The GO and KEGG analyses revealed 23 differentially expressed mRNAs that were enriched in terms and pathways related with ribosomal proteins (RPs).

Ribosomes are subcellular organelles composed of two different subunits^17^, and each subunit contains various numbers of ribosomal RNAs (rRNAs) and RPs. Large 60S ribosomal subunits assemble with small 40S subunits to form 80S ribosomes. In mammals, the 80S ribosomal nucleoprotein complex contains 4 rRNAs and about 80 proteins, with more than 150 associated proteins and about 70 small nucleolar RNAs^18^. The small 40S subunit mediates the interactions between tRNAs and mRNA and selects the correct tRNA for the decoding centre. The large 60S subunit harbours the peptidyl transferase centre and provides the exit tunnel for the growing nascent polypeptide chain. Ribosomes function in translating mRNAs into proteins and translation is tightly depended on the ribosome proteins (RPs)^19^. RPs are highly conserved, so quantitative deficiencies result in reduced protein synthesis^20^, which can affect a range of pathological processes such as cancer^21^, genetic diseases^22^, and viral infection^23^.

RP-encoding genes are widely dispersed. Both human sex chromosomes and the autosomes (all but chromosomes 7 and 21) carry one or more RP genes^20^. Disturbance in translational homeostasis was shown to be involved in the pathogenesis of neurodegenerative disorders^24,25^. For example, a decline in the amount of rRNA was found to be associated with the progression of Alzheimer’s disease^26^. Ribosomes may not be involved only in severe psychiatric disorders. For example, the copy numbers of ribosomal genes were shown to increase in schizophrenia and decrease in autism^27^. Mutations in the RP-encoding gene *RPL10* were reported in people with autism^28^, but another study did not find changes in *RPL10* expression associated with autism^29^. Changes in ribosomes have also been associated with depression. The transcriptional activity of ribosomal DNA was diminished in the argyrophilic nucleolar organizer region of brain tissue of patients with MDD, which suggested hypoactivity of neurons in MDD^30^, and another study revealed over-expressed RPs in the hippocampus of a mouse model of MDD^31^. In the current study, we found the mRNAs that encoded RPs were down-regulated, which may decrease the number of ribosomes and subsequently reduce protein synthesis. The down-regulation of mRNAs encoding some RPs may only reduce protein synthesis, which is not as drastic as the complete mutation or deletion of an RP gene. This, combined with other unknown factors, potentially could produce the symptoms of OCD.

Members of the zinc finger protein (ZNF) family have DNA- and RNA-binding motifs and the amino acids are folded into a single structural unit around a zinc atom^32^. ZNF proteins have a wide-range of functions, including transcription and DNA recognition^33^. *ZNF804A* has been identified as one of the most compelling risk genes associated with psychiatric disorders^34,35^. In the current study, we found that the *ZNF721* mRNA was down-regulated in the OCD patients.

COMMD (copper metabolism domain containing) proteins (also known as MURR1) were discovered about 10 years ago, and 10 COMMD proteins are known so far. They are involved in, for example, copper homeostasis, regulating transcription factor NF-κB (nuclear factor κB), and cell proliferation^36^. COMMD6, a ubiquitously expressed small soluble protein and endogenous inhibitor of NF-κB, binds DNA and activates transcription^37,38^. Activation of NF-κB has been associated with some neurodegeneration diseases as consequences of the neurotoxic role of NF-κB^39^. The down-regulation of *COMMD6* or the action of another NF-κB inhibitor NFKBIA may increase the activation of NF-κB, which might impair the function of the hippocampus in individuals with OCD.

NADH dehydrogenase (ubiquinone) 1 alpha subcomplex (NDUFA4) is the 14th subunit of cytochrome c oxidase. NDUFA4L2 inhibits complex I of oxidative phosphorylation, which is the final oxygen-accepting enzyme complex of the mitochondrial respiratory chain, to mediate a shift to glycolysis in growing cells and cancer tissues^40^. The over-expression of *NDUFA4* seen in lung cancer cells is in contrast to its down-regulation in Alzheimer’s disease. In a previous genome-wide study, *NDUFA4* was found to be associated with Alzheimer’s disease and was identified as a potential biomarker of the disease^41^. Ubiquinol cytochrome c reductase binding protein (UQCRB) is important for mitochondrial complex III stability, electron transport, cellular oxygen sensing, and angiogenesis. *NDUFA*, *COX7C*, and *UQCRB* are involved in the mitochondrial respiratory chain, and all three were down-regulated in the OCD patients. However, there is limited knowledge about the relationship between mitochondrial dysfunction and OCD.

The genes encoding type-2 bitter-taste receptors (TAS2R30 and TAS2R46) were up-regulated in OCD. TAS2Rs are expressed widely outside the brain, but their relationship to OCD is not known.

CACNB4 is one of the voltage-gated calcium channel beta subunits, which was recently found to function in neuronal excitability and gene transcription^42^. *CACNB4* was over-expressed in schizophrenia and was associated with depressing the calcium currents that drive spine formation and stabilization, and increased *CACNB4* expression was found to drive small spine loss^43^. We consider the up-regulation of *CACNB4* detected in our study may be related with the pathogenesis of OCD.

Several limitations in our study should be noted. First, the sample size was relatively small, which may have reduced the statistical power of the comparison of gene expression between the OCD and healthy control groups. There were inconsistencies in the direction of gene alterations between the microarray analysis and the qRT-PCR validation, likely because different samples were used for validation and the patients were at different stages of the disorder and under different treatment regimes.

In conclusion, we detected altered gene expression patterns in patients with OCD and highlighted the role of RP genes in the pathogenesis of OCD.

## Materials and Methods

### Participant profiles

This study was conducted in the Wuxi Mental Health Centre of Nanjing Medical University, Wuxi, Jiangsu Province, China. Thirty patients with OCD and 30 sex- and age-paired healthy controls were recruited. There were 20 males and 10 females in both groups. The mean age was 28.8 ± 12.0 years (range 15–60 years) and 28.8 ± 11.1 years (range 17–56 years) for the patient and the control groups respectively. The diagnosis of OCD was confirmed using the structured clinical interview for DSM-IV disorders (SCID). Patients with schizophrenia, MDD, comorbid axis I disorder, or with a history of neurological disease were excluded. Healthy controls who were free from any psychiatric illness or major medical condition were recruited from the local community.

This study was approved by the human ethics committee of the Wuxi Mental Health Centre of Nanjing Medical University. Written informed consent was provided by each participant. All study procedures were in accordance with the Helsinki Declaration of 1975.

### Blood sample collection and PBMC isolation

Peripheral blood was collected in 10-ml vacutainer tubes containing EDTA and immediately stored at 4 °C. Whole blood was processed within 2 h of collection.

Ficoll density gradient centrifugation was used to separate the peripheral blood mononuclear cells (PBMCs). Briefly, saline diluted blood was layered over Ficoll, then centrifuged to separate red blood cells, PBMCs, and plasma. The PBMCs were gently and entirely sucked up from the layer of Ficoll and transferred to a new tube, which was washed twice.

### Total RNA isolation

Total RNA was extracted from the PBMCs using TRIzol reagent (Invitrogen, USA) according to the manufacturer’s instructions and quantified using a NanoDrop ND-2000 (Thermo Scientific). RNA integrity (RIN) was assessed using an Agilent Bioanalyzer 2100 (Agilent Technologies).

The mean (SD) RIN for all the samples was 9.29 (0.48). The 28S to 18S rRNA ratio was 2.79 (0.35) and the RIN was ≥7. For 28S:18S a RIN value ≥0.7 was considered to be within the range of acceptable RNA quality according to the manufacturer’s instructions.

### mRNA microarray, labelling, hybridization, and scanning

Total RNA was labelled with a mRNA Complete Labelling and Hyb Kit (Agilent Technologies) and hybridized on a Human lncRNA Microarray 4.04 × 180 K (Agilent Technologies). The microarray contains 30,656 probes for human mRNA, all of which were derived from authoritative databases, including RefSeq Build, Ensemble Release, GenBank, and Unigene Build. Total RNA (200 ng each) was reverse transcribed to double-strand cDNA, then synthesized into cRNA and labelled with cyanine-3-CTP. The labelled cRNAs were hybridized to the microarray. After washing, the arrays were scanned using an Agilent Microarray Scanner (G2505C, Agilent Technologies).

### Validation by qRT-PCR

Total RNA was isolated from PBMCs from another 26 pairs of OCD and healthy controls using TRIzol reagent (Invitrogen) with on-column DNase I treatment as described by the manufacturer. cDNA was synthesized using a High Capacity RNA-to-cDNA Kit (Invitrogen) according to the manufacturer’s instructions. The qRT–PCRs were performed using the primers listed in Table 4 and SYBR^®^ Select Master Mix (Invitrogen) on a 7900HT real-time PCR machine (Applied Biosystems, USA) with the following cycles: 2 min at 50 °C, 2 min at 95 °C, then 40 cycles of 15 s at 95 °C, 60 s at 60 °C, followed by a standard dissociation protocol to ensure that each amplicon was a single product. All quantifications were normalized to *ACTB*. The qRT–PCRs were performed in triplicate for each independent sample.

**Table 4:** Primers for the differently expressed mRNAs used in the qRT-PCRs.

	Gene Symbol	Primer	Primer sequences
1	RPS7	1-Forward	atgttcagttcgagcgcc
		1-Reverse	ttcgcgtactagccggac
2	RPS3A	2-Forward	tggcatggatcttacccg
		2-Reverse	gatttggcggacctgttg
3	RPL34	3-Forward	tgacaggatcaagcgtgc
		3-Reverse	ttgcagcatttgctgagg
4	RPS24	4-Forward	cgccatcatgaacgacac
		4-Reverse	gccagttgtcttgccacc
5	RPL23	5-Forward	acagacttcccgctgctg
		5-Reverse	aatcatgcaatgctgcca
6	RPL41	6-Forward	gaggccacaggagcagaa
		6-Reverse	agaggaccaacatgggca
7	RPL7	7-Forward	gcgaaggaatttcgcaga
		7-Reverse	ttgccagcttttcttgcc
8	RPL26	8-Forward	cttccgaccgaagcaaga
		8-Reverse	ctggggtgaatgcctacg
9	ZNF721	9-Forward	tggacggtacacagccct
		9-Reverse	caaaggctctgccacgat
10	COMMD6	10-Forward	ggcaatcagaagagtgaggc
		10-Reverse	tcgtctttccaactctgcg

### Data analysis

Agilent Feature Extraction software (version 10.7.1.1; Agilent Technologies) was used to analyse the array images to obtain the raw data. GeneSpring (GX v11.5.1 software package; Agilent Technologies) was employed to analyse the raw data. The raw data were first normalized with the quantile algorithm, followed by differential expression analysis using a student t-test. The probes that had at least 1 out of 2 conditions and had 75% flags in “P” were chosen for further data analysis. Differentially expressed genes were identified based on fold change as well as the *p-*value calculated with the student t-test. The threshold set for up- and down-regulated genes was fold change ≥ 1.5 and false discovery rate ≤ 0.05.

The expression levels of mRNAs between the OCD patients and healthy controls were analysed using the Mann-Whitney U test.

To correlate the differentially expressed mRNAs with biological processes, we annotated the mRNAs with GO terms and KEGG pathways (http://www.genome.ad.jp/kegg/) to determine their potential roles. Then, we performed a hierarchical clustering analysis to display the distinguishable gene expression patterns between the OCD and healthy groups. The lower the *p*-value, the more significant the correlation; the recommended *p*-value cut-off was 0.05.

## Data Availability

All the microarray data have been deposited in the Gene Expression Omnibus (GEO) at the NIH National Centre for Biotechnology Information under Series Number GSE78104.
